# Predictors of Cyclophosphamide Resistance in Granulomatosis with Polyangiitis: A Retrospective Cohort Study

**DOI:** 10.31138/mjr.150736.pcr

**Published:** 2026-03-01

**Authors:** Pınar Akyüz Dağlı, Esra Kayacan Erdoğan, Hatice Ecem Konak, Bahar Özdemir Ulusoy, Serdar Can Güven, Berkan Armağan, Rezan Koçak Ulucaköy, Kevser Orhan, Ebru Atalar, İsmail Doğan, Yüksel Maraş, Ahmet Omma, Orhan Küçükşahin, Şükran Erten, Hakan Babaoğlu

**Affiliations:** 1 Clinic of Rheumatology, Ministry of Health Ankara Bilkent City Hospital, Ankara, Türkiye;; 2 Clinic of Rheumatology, Ankara Gaziler Physical Therapy and Rehabilitation Training and Research Hospital, Ankara, Türkiye;; 3 Division of Rheumatology, Department of Internal Medicine, Ankara Yıldırım Beyazıt University, Ankara Bilkent City Hospital, Ankara, Türkiye;; 4 University of Health Sciences, Division of Rheumatology, Department of Internal Medicine, Ankara City Hospital, Ankara Bilkent City Hospital, Ankara, Türkiye

**Keywords:** cyclophosphamide, granulomatosis with polyangiitis, ANCA-associated vasculitis, remission induction, induction failure, treatment resistance

## Abstract

**Objective:**

Cyclophosphamide remains a cornerstone of remission induction therapy in granulomatosis with polyangiitis (GPA); however, the presence of resistant patients necessitating a switch to alternative agents, such as rituximab, remains a clinical challenge. Identifying predictors of cyclophosphamide resistance could improve patient stratification and optimise treatment strategies.

**Methods:**

This retrospective cohort study included 75 patients diagnosed with GPA and treated at Ankara Bilkent City Hospital between 2018 and 2023.Clinical and laboratory data were extracted from electronic medical records. Baseline characteristics, organ involvement, and serological profiles were compared between cyclophosphamide-responsive and cyclophosphamide-resistant patients. Logistic regression analysis was performed to identify independent predictors of treatment resistance.

**Results:**

75 patients with GPA analysed. The mean age was 46.1years (SD=14.0).56 patients received cyclophosphamide as first-line therapy, of whom 34 (60.7%) achieved remission, while 22 (39.3%) switched to rituximab. Of these, 17 patients (30.4%) were classified as cyclophosphamide-resistant, while 5 patients (8.9%) switched due to other reasons. Younger age was a significant predictor of cyclophosphamide resistance (OR=0.915, 95%CI:0.856–0.977, p=0.008). The presence of arthritis showed a trend toward association (OR=5.191, 95%CI:0.960–28.065, p=0.056) but did not reach statistical significance. No significant differences were observed in gender, ANCA subtypes, major organ involvement, or comorbidity burden between groups.

**Conclusion:**

Our findings suggest that younger age is associated with a higher likelihood of cyclophosphamide resistance in GPA, potentially indicating a more aggressive disease course. Although arthritis showed a potential association with resistance, further studies are needed to confirm its role.

## INTRODUCTION

Granulomatosis with polyangiitis (GPA) is a disease characterised by necrotising vasculitis of small blood vessels and granulomatous inflammation and is classified under ANCA-associated vasculitides (AAV).^1^ In recent years, great advances have been made in the management of AAV. Treatment with a combination of glucocorticoids and rituximab (RTX) or cyclophosphamide (CYC) is recommended for induction of remission in patients with new-onset or relapsing GPA with organ-or life-threatening manifestations.^2–4^

The selection of therapy should be individualised based on factors such as prognosis, serologic profile, disease severity and activity, comorbidities, and other patient-specific factors.^5^ The data available in the literature is quite limited. Historically, CYC was the primary agent used for remission induction in severe disease; however, recent studies have demonstrated the efficacy of RTX as an alternative. Although CYC plays a pivotal role in remission and patient survival, it is not effective in all patients and is associated with toxicity.^4^ Notably, the RAVE trial demonstrated that while CYC achieved remission in 64% of patients at 6 months, RTX was found to be equally effective.^6^ Additionally, long-term use of CYC has been associated with with malignancy, hematological and infectious side effects.^5^ Consequently, various strategies have been explored to minimise CYC exposure. Failure of remission induction not only increases the risk of disease-related disability but also prolongs exposure to CYC, compounding the risks of cumulative toxicity.^7^ Furthermore, persistent active disease despite first-line therapy is associated with significant mortality and long-term morbidity, including chronic renal failure and pulmonary damage.^8^

The RITUXVAS and MAINRITSAN trials, have demonstrated that RTX is associated with a lower risk of infections, lacks gonadotoxicity, and is particularly effective in preventing relapses.^9,10^ Additionally, data suggest that RTX may be especially beneficial in reducing relapse risk in PR3-ANCA-positive patients.^11^ These findings highlight the importance of individualised treatment selection based on clinical and serological profiles. Both CYC and RTX are now established as viable induction therapies, but determining which treatment is more effective for a given patient remains a crucial question, particularly given the limited therapeutic options.

Identifying the clinical differences between CYC-resistant and CYC-responsive patients is crucial for developing personalised treatment approaches. Understanding these clinical variations could allow for more personalised treatment strategies, ultimately improving disease control and reducing morbidity and mortality with early, effective therapy. Specifically, we aim to investigate whether certain clinical or serological profiles are associated with CYC resistance, helping to predict which patients may benefit more.

## METHOD

### Study Design and Population

This retrospective cohort study included 75 patients diagnosed with GPA who were treated at Ankara Bikent City Hospital between January 1, 2018, and December 31, 2023. Inclusion criteria required a clinically confirmed diagnosis of GPA in patients aged 18 years or older. Patients were excluded if they had incomplete medical records and unverified diagnoses.

Patient Data were extracted from electronic medical records using a standardised data collection form by researchers blinded to the study hypotheses. Collected data included demographic details, laboratory findings, and comorbidities. Additionally, organ involvement was systematically assessed according to the BVAS (Birmingham Vasculitis Activity Score) domains.^12^ The study was conducted and reported in accordance with the STROBE (Strengthening the Reporting of Observational Studies in Epidemiology) guidelines.

The medical records of GPA patients were analysed, focusing specifically on the first remission induction period, with particular attention to those who received CYC for remission induction and the decisions to switch to RTX during this phase. All patients received monthly intravenous cyclophosphamide at a dose of 15 mg/kg, following the standard protocol adapted from the CYCLOPS trial.^13^ We recorded the timing of the switch (month of induction) and the reasons for CYC cessation. CYC resistance was defined as the failure to achieve remission, determined by the treating physician based on objective evidence of persistent or worsening disease activity. This definition aligns with EULAR recommendations, which describe refractory disease as no improvement or worsening of signs, symptoms, or other features of active AAV despite standard induction therapy.^2^ Patients who switched therapy due to side effects or other reasons related to drug intolerance were not classified as resistant. First, we compared CYC-responsive and CYC-resistant patients in terms of organ involvement, serological profile, and demographic characteristics to identifty differences between the groups. Then, to determine independent predictors of CYC resistance, we performed logistic regression analysis.

The study adhered to the principles of the Declaration of Helsinki and was approved by the Institutional Review Board of Ankara Bilkent City Hospital (E1-23-3801). Due to the retrospective design, informed consent was waived. Patient confidentiality and data anonymity were maintained throughout the study.

### Statistical Analysis

Statistical analyses were performed using SPSS Statistics for Windows, Version 21 (IBM Corp., Armonk, NY). Continuous variables were summarised as means and standard deviations (SD) or medians and interquartile ranges (IQR) as appropriate, while categorical variables were presented as frequencies and percentages. The normality of continuous variables was assessed using the Shapiro-Wilk test. For normally distributed variables, comparisons between groups were performed using the independent samples t-test. Non-normally distributed variables were compared using the Mann-Whitney U test. Categorical variables were compared between cyclophosphamide-responsive and -resistant groups using the Chi-square test or Fisher’s exact test where appropriate. Variables with a p-value of less than 0.1 in univariate analysis were included in a multivariate logistic regression model to identify independent predictors of resistance to cyclophosphamide treatment. A two-stage approach was used to select variables for the multivariate logistic regression model. An initial univariate screening was performed using a p-value threshold of < 0.1. This lenient criterion was intentionally chosen to prevent the premature exclusion of important confounding variables or predictors that might only demonstrate significance in the multivariate context, thereby mitigating the risk of a Type II error. The logistic regression results were expressed as odds ratios (OR) with 95% confidence intervals (CI). A p-value of less than 0.05 was considered statistically significant.

## RESULTS

A total of 75 patients with GPA were included in the study. The mean age at diagnosis was 46.1 years, and 54.7% of the patients were male (**Table 1**). A history of smoking was present in 29.3% of the cases. The most common comorbidities were hypertension (30.7%) and diabetes mellitus (16%). The most frequently observed clinical manifestations were pulmonary involvement (72%), followed by ear, nose, and throat (53.3%) and renal involvement (42.7%). The majority of patients were PR3-ANCA positive (89.3%). The mean BVAS score was 12.3. While 98.6% of all included patients met the 2022 EULAR/ACR classification criteria for GPA, one patient with localised disease was ANCA-negative and received a diagnosis based on histopathological confirmation.

**Table 1.** Demographic, clinical, serological characteristics, and comorbidities.

**Characteristic**	**Mean (SD)/n (%)**
Age at Diagnosis (years), mean	46.1 (14.8)
Gender, male	41 (54.7%)
Smoking Status, ever	22 (29.3%)
Comorbidity	43 (57.3%)
Diabetes Mellitus	12 (16.0%)
Hypertension	23 (30.7%)
Hyperlipidemia	2 (2.7%)
Coronary Artery Disease	6 (8.0%)
Osteoporosis	5 (6.7%)
Other comorbidity	14 (19.0%)
GPA 2022 Criteria Score	8.6 (2.2)
Skin Involvement	10 (13.3%)
Nervous System Involvement	2 (2.7%)
Ocular Involvement	19 (25.3%)
ENT Involvement	40 (53.3%)
Pulmonary Involvement	54 (72.0%)
Alveolar Haemorrhage	11 (14.7%)
Renal Involvement	32 (42.7%)
Pauci-immune glomerulonephritis	20 (26.7%)
ANCA ELISA	
• MPO	4 (5.3%)
• Negative	4 (5.3%)
• PR3	67 (89.3%)
BVAS Score	12.3 (5.5)

* GPA: granulomatosis with polyangiitis; CYC: cyclophosphamide; RTX: rituximab; ENT: Ear; Nose; and Throat; ANCA: antineutrophil cytoplasmic antibody; PR3-ANCA: proteinase 3; MPO: myeloperoxidase; BVAS:Birmingham Vasculitis Activity Score; SD: standard deviation; N: number of patients.

**Figure 1.**
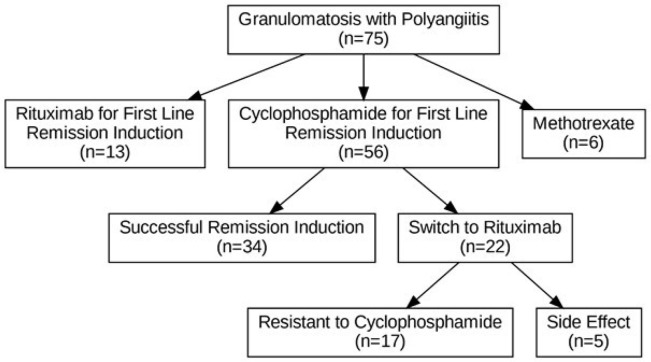
Flowchart of treatment pathways of GPA patients.

Of the 75 patients included in the study, first remission induction therapy consisted of CYC in 56 patients, rituximab RTX in 13 patients, and MTX in 6 patients (**Figure 1**). Among the 56 patients who received CYC as first-line induction therapy, 34 patients (60.7%) achieved remission, while 22 patients (39.3%) required a treatment change. Of these, 17 patients (77.3%) were switched to RTX due to refractory disease, while 5 patients were switched due to adverse effects of CYC. Specifically, 2 patients (9.1%) had side effects, 2 (9.1%) developed infections, and 1 (4.5%) had a suspicion of malignancy. The median time to treatment change from CYC to RTX was 4 months (**Table 2**).

**Table 2.** Median time to treatment change from CYC to RTX and reasons for changes.

**Characteristic**	**GPA (n=22)**
Median Time to Treatment Change, months (min- max)	4 (0–5)
**Reason for Induction Treatment Change, n (%)**	
Side Effect	2 (9.1%)
Refractory Disease	17 (77.3%)
Infection	2 (9.1%)
Suspicion of Malignancy	1 (4.5%)

GPA: granulomatosis with polyangiitis; CYC: cyclophosphamide; RTX: rituximab.

When comparing CYC-resistant and CYC-responsive patients, those in the resistant group were younger at diagnosis (41.2 vs 51.1 years, p=0.015) (**Table 3**). Additionally, arthritis was more prevalent in the resistant group (35.3% vs. 8.8%, p=0.019). There were no significant differences between the groups in terms of gender distribution, smoking history, comorbidities, or ANCA subtypes. BVAS scores were similar. Pulmonary involvement was observed in 73.5% of CYC-responsive patients and 64.7% of resistant patients. Renal involvement rates were comparable (44.1% vs. 47.1%).

**Table 3.** Comparison of cyclophosphamide-sensitive and -resistant patients.

**Characteristic**	**CYC Sensitive (N=34)**	**CYC Resistant (N=17)**	**P^1^ Value**	**Odds Ratio (95% CI)**	**P^2^ value**
Age at Diagnosis, years, mean (SD)	51.1 (13.8)	41.2 (12.1)	0.015	0.915 (0.856–0.977)	0.008
Gender n (%)	19 (55.9%)	12 (70.6%)	0.311		
Smoking, n (%)	13 (38.2%)	8 (47.1%)	0.546		
Comorbidity*	21 (61.8%)	11 (64.7%)	0.838		
Charlson comorbidity index, median (min-max)	2 (0–5)	1 (0–6)	0.137		
GPA 2022 Criteria Score	8.5 (2.2)	9.5 (2.2)	0.134		
Skin Involvement, n (%)	3 (8.8%)	3 (17.6%)	0.213		
Ocular Involvement, n (%)	10 (29.4%)	3 (17.6%)	0.323		
ENT Involvement, n (%)	21 (61.8%)	9 (52.9%)	0.546		
Pulmonary Involvement, n (%)	25 (73.5%)	11 (64.7%)	0.514		
Alveolar Hemorrhage, n (%)	5 (14.7%)	1 (5.9%)	0.357		
Renal Involvement, n (%)	15 (44.1%)	8 (47.1%)	0.842		
Pauci-immune glomerulonephritis, n (%)	9 (26.5%)	7 (41.2%)	0.286		
Arthritis, n (%)	3 (8.8%)	6 (35.3%)	0.019	5.191 (0.960–28.065)	0.056
ANCA ELISA, n (%)					
• MPO	2 (5.9%)	0 (0.0%)			
• Negative	2 (5.9%)	2 (11.8%)	0.472		
• PR3	30 (88.2%)	15 (88.2%)			
BVAS Score	12.4 (5.4)	12.6 (6.2)	0.863		

*GPA: granulomatosis with polyangiitis; CYC: cyclophosphamide; RTX: rituximab; ENT: Ear; Nose; and Throat; ANCA: antineutrophil cytoplasmic antibody; PR3-ANCA: proteinase 3; MPO: myeloperoxidase; BVAS: Birmingham Vasculitis Activity Score; SD: standard deviation; N: number of patients; P1: Univariate p-value; P2: Multivariate p-value.

The logistic regression model demonstrated moderate explanatory power with a Nagelkerke’s R^2^ of 0.221 and a reasonable fit. Younger age was a significant predictor (OR = 0.915, 95% CI: 0.856–0.977, p = 0.008). The presence of arthritis showed a trend toward association (OR = 5.191, 95% CI: 0.960–28.065, p = 0.056).

## DISCUSSION

In this study, we present retrospective comparative data on CYC induction failure in GPA. The overall response rate to CYC induction therapy was approximately 60%. Younger age emerged as the sole statistically significant predictor of treatment resistance. Although a trend toward association was observed with arthritis, this did not reach statistical significance. Notably, no significant differences were found for established markers such as ANCA subtype or major organ involvement. These results underscore the heterogeneity of treatment response in GPA and suggest that resistance may be driven by factors beyond standard clinical characteristics.

Published CYC induction response rates in GPA range from 60% to 80%, contingent on disease severity and study design. Our observed response rate of approximately 60% aligns with the lower end of this range but is consistent with major clinical trials, including the EUVAS and RAVE studies. The RAVE trial reported a CYC remission rate of 64% at six months,^6^ comparable to our results. Similarly, data from EUVAS trials indicated that CYC achieves remission in approximately 60–70% of patients, with variations based on treatment protocols and follow-up duration. The need for treatment escalation in 40% of our cohort highlights the limitations of CYC in certain subsets and underscores the necessity for improved predictive markers for personalised GPA management. Notably, our observed rate of CYC resistance (30.3%) is higher than reported in large cohorts such as the French Vasculitis Group study, which documented only 51 cases of induction failure over 15 years.^7^ This discrepancy may be partly explained by differences in cohort size, referral patterns to tertiary centers, and definition of resistance (clinical decision to switch vs. strictly induction failure). Furthermore, our single-center cohort included a higher proportion of younger patients, who may be predisposed to refractory disease.

Age is a critical factor in GPA, influencing disease severity, treatment response, and prognosis.^14–16^ Our study identified a significant association between younger age and CYC resistance, suggesting a more aggressive or refractory disease course in this demographic. This observation aligns with findings in other autoimmune diseases where younger age is linked to heightened immune activation. However, our results contrast with a key report from the French Vasculitis Study Group, which identified older age as a predictor of treatment resistance.^17^ One possible explanation for this discrepancy is that older patients often receive less aggressive immunosuppressive therapy due to concerns about treatment-related toxicity, infections, and comorbidities, leading to an apparent association between age and resistance in some studies. In our study, although patients in the resistant group were significantly younger, the prevalence of severe organ involvement, serological markers, and comorbidites were similar, suggesting that other factors may contribute to CYC resistance in younger patients. Previous studies have primarily associated older age with increased mortality and higher infection risk rather than treatment. For example, in a long-term follow-up study of AAV patients, older age was associated with higher mortality but not necessarily with treatment failure.^8^ Additionally, In elderly patients with AAV, poorer clinical outcomes are primarily attributed to age-related immunosenescence and treatment-related toxicity rather than differences in disease activity. Research suggests that aging weakens the immune system, making older patients more vulnerable to the adverse effects of immunosuppressive therapy. Haris et al. found that while a reduced-dose immunosuppressive regimen in elderly AAV patients was as effective as standard doses in younger patients, overall mortality was significantly higher, primarily due to cardiovascular complications rather than vasculitis or infection.^18^ Similarly, Judge et al. reported that despite receiving lower cumulative doses of CYC, elderly AAV patients had higher rates of leukopenia, infections, and dialysis dependency at presentation, all of which significantly increased mortality risk.^19^ Given these conflicting findings on age as a predictor of resistance, its role requires clarification in future prospective studies.

Although data on the relationship between gender and treatment resistance in GPA are limited, some studies suggest a potential association. In a community-based cohort of patients with AAV (including MPA, GPA, and renal-limited vasculitis), treatment resistance was observed in 23% of 334 treated patients, with female gender and black ethnicity identified as independent predictors of CYC resistance.^20^ Similarly, another study on AAV patients reported that female gender was associated with a higher likelihood of treatment resistance.^21^ However, contrary to these findings, our study did not observe a significant difference in treatment resistance between male and female patients, similar to the French Vasculitis Study Group,^17^ suggesting that gender may not be a consistent predictor of CYC response in all populations. Further large-scale, multicenter studies are needed to clarify the potential role of gender in GPA treatment outcomes.

In addition to disease status, comorbidities may play a role in treatment response and outcomes in GPA. Comorbid conditions can affect treatment efficacy in several ways: limitations on immunosuppressive therapy due to toxicity concerns or worsening of comorbid conditions due to immunosuppressive treatment itself. These factors create significant challenges in the induction treatment process and may contribute to poor outcomes.A cohort study from Denmark demonstrated that GPA patients with pre-existing comorbidities had significantly higher mortality in the early follow-up period compared to those without comorbidities.^22^ In our study, although we did not assess mortality outcomes, comorbidities were found similar between the groups. The literature presents conflicting evidence regarding organ involvement as a predictor of CYC resistance. Some studies link high disease activity and baseline organ damage to treatment failure.^23^ Specifically, granulomatous features such as orbital masses and tracheobronchial involvement have been associated with a refractory course where RTX may be more effective.^24–26^ However, we could not demonstrate similar findings in our study, potentially due to a limited number of cases. Another report has associated PR3-ANCA positivity and relapsing disease with CYC induction failure.^7^ Furthermore, severe renal disease,^20^ initial renal disease,^21^ and elevated serum creatinine levels^27,28^ have been found to be associated with treatment resistance in other studies. Despite conflicting evidence, the efficacy of CYC in renal involvement has been well-documented, and there is insufficient data to suggest that renal involvement itself should be used as a predictor of CYC resistance. Rather, severe necrotising glomerulonephritis and high baseline creatinine levels may be more indicative of poor prognosis than resistance to therapy.^29^ Furthermore, in the pre-randomisation phase of the WEGENT study, which analysed patients with GPA and MPA who failed to achieve remission with corticosteroids and IV CYC, solely alveolar hemorrhage was independently associated with induction-resistant disease.^28^ In our study, arthritis was more frequently observed in the CYC-resistant group, although this association did not reach statistical significance in logistic regression analysis. We did not identify any additional organ involvement that could reliably predict CYC resistance.

A key strength of this study is its presentation of real-world comparative data on CYC induction failure in GPA, a relatively underexplored topic. Nevertheless, several limitations warrant consideration. First, the data is retrospective. Second, the modest sample size, particularly of the CYC-resistant group, limited the statistical power to identify predictors with smaller effect sizes and precluded a meaningful subgroup analysis of specific manifestations.

In conclusion, this study identifies younger age as a significant independent predictor of CYC resistance in patients with GPA. By identifying younger age as a significant predictor of resistance, our findings add to the current understanding of heterogeneity in treatment response and highlight a potentially high-risk subgroup that may benefit from early alternative induction strategies such as RTX.While a trend toward resistance was observed with arthritis, a significant association with traditional risk-stratification markers, such as ANCA subtype or major organ involvement, was not found. This suggests that the mechanisms of treatment failure may be driven by more subtle immunological or genetic factors that are not captured by standard clinical assessments. Therefore, elucidating these underlying pathways and validating our findings in larger, prospective cohorts are critical next steps toward personalising induction therapy and improving long-term outcomes in GPA.

## Data Availability

The data supporting the results of this study are available from the corresponding author upon reasonable request.

## NOTES

The authors note that this study was previously presented in abstract form as *“Identification of Independent Predictors of Cyclophosphamide Resistance in Patients with Granulomatosis with Polyangiitis”* at the *17th Anadolu Rheumatology Days*, 5–9 June 2024, Kyrenia, Turkish Republic of Northern Cyprus.
